# Glial cell crosstalk in Parkinson's disease: Mechanisms, implications, and therapeutic strategies

**DOI:** 10.1016/j.fmre.2024.12.023

**Published:** 2025-01-11

**Authors:** Ning Wang, Xiao Xiao, Zilu Chen, Keyang Xu, Xiaoyi Cao, Dandan Kou, Jianfeng Zeng

**Affiliations:** Center for Molecular Imaging and Nuclear Medicine, State Key Laboratory of Radiation Medicine and Protection, School for Radiological and Interdisciplinary Sciences (RAD-X), Collaborative Innovation Center of Radiological Medicine of Jiangsu Higher Education Institutions, Soochow University, Suzhou 215123, China

**Keywords:** Parkinson's disease, Neuroinflammation, Microglia-astrocyte crosstalk, Glial Cells, Neurodegeneration

## Abstract

Parkinson's disease (PD) is a prevalent age-related neurodegenerative disorder marked by the progressive loss of dopaminergic neurons and the formation of Lewy bodies. Traditionally viewed as primarily a dopaminergic disorder, recent research has highlighted the significant role of neuroinflammation and the complex interplay between glial cells, particularly astrocytes and microglia, in PD pathogenesis. This review explores the multifaceted roles of astrocytes, microglia and oligodendrocytes in PD, focusing on their involvement in maintaining glutamate and ion homeostasis, energy metabolism, and the inflammatory response. We discuss the dual nature of these glial cells, which can both support and harm neuronal health under different conditions. We also examine the molecular mechanisms underlying glial cell communication, including cytokines, chemokines, extracellular vesicles, gap junctions, and neurotransmitter systems. Finally, we propose potential therapeutic strategies targeting these glial interactions to modulate neuroinflammation and protect neurons, offering new avenues for PD treatment. Understanding the diverse functions and interactions of glial cells in the central nervous system is crucial for developing effective interventions for PD and other neurodegenerative diseases.

## Graphical abstract

**Figure:**
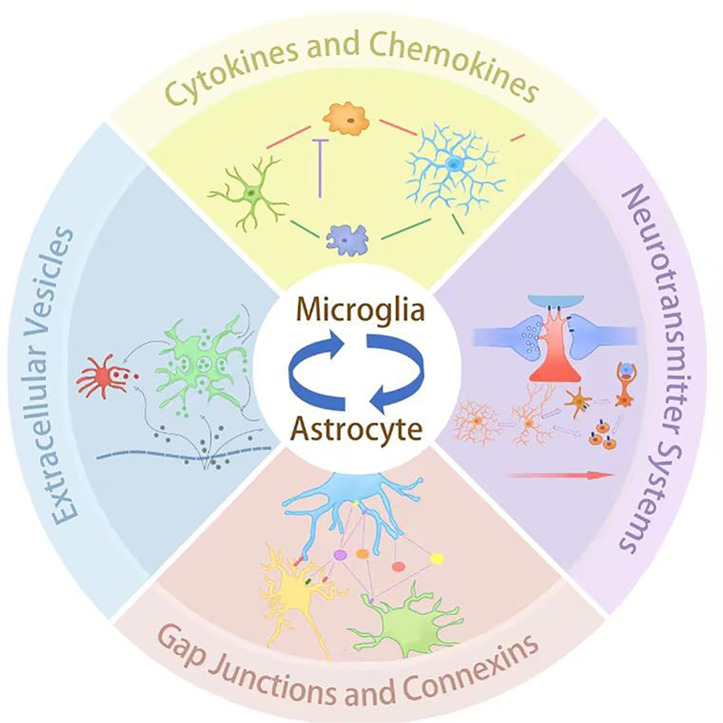

## 1 Introduction

Parkinson's disease (PD) is the second most prevalent age-related neurodegenerative disorder globally, affecting approximately 1%−2% of individuals over the age of 65 [1]. It is characterized by the progressive loss of dopaminergic neurons in the substantia nigra pars compacta and the accumulation of α-synuclein in the Lewy bodies [2]. PD manifests through motor symptoms such as resting tremor, bradykinesia, and muscular rigidity, as well as non-motor symptoms including sleep disturbances, gastrointestinal dysfunction, and cognitive deficits [3]. Although clinical attention often focuses on these symptoms, the pathogenesis of PD is complex and involves a multifaceted interplay of genetic, environmental, and molecular factors [4].

Recent advancements in PD research have begun to challenge traditional understanding of the disease. Novel insights into the genetic underpinnings reveal that while 5%−10% of PD cases are hereditary, involving mutations in genes such as SNCA, PRKN, PINK1, DJ-1, and LRRK2 [[5], [6], [7]], the majority are idiopathic, hinting at a broader spectrum of contributory factors beyond genetics alone. This includes emerging evidence on the role of the gut-brain axis, environmental toxins, and even viral infections in the disease's etiology, suggesting a more expansive view of PD pathogenesis [8].

**Fig. 1:**
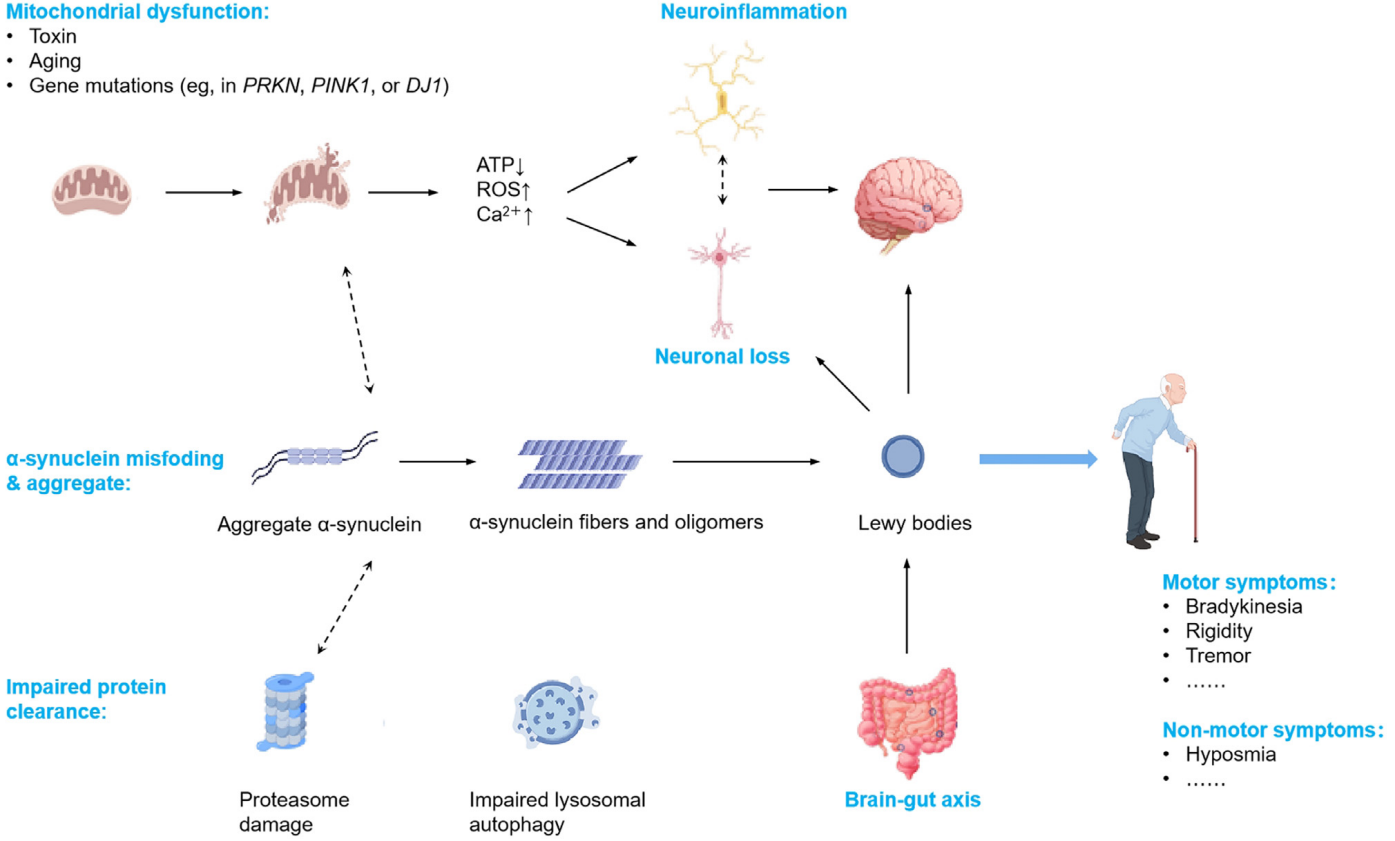
**Pathogenic mechanisms underlying PD.** This schematic illustrates the multifaceted molecular mechanisms contributing to PD. Key factors include mitochondrial dysfunction, impaired protein clearance via proteasome and lysosomal pathways, and neuroinflammation, all leading to the misfolding and aggregation of α-synuclein. Aggregates of α-synuclein form fibrils and oligomers, contributing to the formation of Lewy bodies, a hallmark of PD. Additionally, disruptions in the brain-gut axis and oxidative stress (increased ATP, ROS, and Ca^2+^ levels) exacerbate neuronal loss. These molecular abnormalities culminate in both motor symptoms (bradykinesia, rigidity, and tremor) and non-motor symptoms (e.g., hyposmia), characteristic of PD pathology.

Moreover, the traditional view of PD as merely a dopaminergic disorder is being supplemented by recognition of its systemic nature, implicating a range of cellular processes such as protein misfolding, mitochondrial dysfunction, oxidative stress, and particularly, neuroinflammation (Fig. 1) [9,10]. The latter has gained prominence, with inflammation and inflammatory immunity proposed as significant contributors to PD. This paradigm shift suggests that chronic inflammation represents a critical imbalance between pro-inflammatory and anti-inflammatory processes, with sustained inflammatory stress leading to dysregulation and compromised immune responses.

Central to this discussion is the role of glial cells, including astrocytes, microglia and oligodendrocytes, which, far from their once-presumed supportive roles, are now recognized as key players in the disease's pathogenesis [11,12]. The intricate molecular dialogue between these cells coordinates a range of processes from brain development to the manifestation of neurofunctional disorders. Recent studies have explored the mutual influence of astrocytes, microglia and oligodendrocytes in PD, highlighting their potential as targets for glia-based functional therapies [[13], [14], [15], [16]]. This review aims to delve into the complex interplay between glial cells within the context of PD, striving for a better understanding of their communication mechanisms. By integrating recent advancements and challenging traditional paradigms, we hope to illuminate new pathways for research and therapeutic intervention in PD.

## 2 Role and mechanisms of glial cells in Parkinson's disease

### 2.1 Astrocytes

The term “Astroglia” was first coined in the 19th century by Rudolf Virchow, marking the initial recognition of these cells’ existence within the central nervous system (CNS). A decade following Virchow's description, Camillo Golgi advanced our understanding by employing silver chromate staining techniques, which allowed for the detailed visualization of astrocytes’ morphology. This methodological breakthrough led to the conceptualization of glial cells as the “glue” of the brain [17], a term that reflects their perceived primary function at the time. In PD, pathological stimuli such as α-synuclein can induce astrocytes to adopt the A1 phenotype under the influence of cytokines secreted by M1 microglia. This process involves significant morphological, transcriptional, and functional changes. A1 reactive astrocytes upregulate the expression of pro-inflammatory cytokines (TNF-α, IFN-γ, IL-1β) and neurotoxic factors such as lipocalin-2 (LCN2) via the NF-κB signaling pathway, exacerbating neurodegeneration and contributing to disease progression [[18], [19], [20]]. Conversely, anti-inflammatory cytokines, such as IL-4, IL-10, and IL-13, released by M2 microglia, can drive astrocytes toward the A2 phenotype. These A2 astrocytes promote neuronal survival and repair by activating the JAK/STAT3 signaling pathway, leading to the secretion of neurotrophic and anti-inflammatory factors such as vascular endothelial growth factor (VEGF), brain-derived neurotrophic factor (BDNF), transforming growth factor-β (TGF-β), and IL-10. These factors support neuronal growth, survival, and regeneration, demonstrating the dual roles astrocytes play in neuroinflammation [21]. However, the A1/A2 framework oversimplifies the complex and heterogeneous nature of astrocytes. Recent research has revealed that astrocyte subpopulations exhibit distinct transcriptomic and proteomic profiles, with their functional roles influenced by regional, temporal, and pathological contexts. This underscores the importance of moving beyond binary classifications to understand the diverse and dynamic roles of astrocytes in PD.

Astrocytes exhibit significant heterogeneity, with their location and interactions within the CNS regulated by developmental programs and stimulus-specific cellular responses [22]. This heterogeneity plays a crucial role in PD, which is characterized by the selective degeneration of dopaminergic neurons in the substantia nigra (SN), whereas neurons in the adjacent ventral tegmental area (VTA) are relatively spared. The differential vulnerability between these regions is partly attributed to astrocytic heterogeneity [23]. Astrocyte density is higher in the VTA than in the SN, highlighting regional heterogeneity. Additionally, astrocytes in these regions exhibit functional heterogeneity [24]. For instance, astrocytes in the VTA express higher levels of the neuroprotective factor growth/differentiation factor 15 (GDF15) compared to those in the SN, providing enhanced protection for VTA dopaminergic neurons against PD-related toxicity [25]. Beyond their foundational structural role, astrocytes are now recognized for their critical functions in maintaining glutamate and ion homeostasis, metabolizing cholesterol and sphingolipids, and modulating the brain's response to environmental stressors [26,27]. These capabilities underscore their importance in maintaining neural health and highlight their significant impact on the pathophysiology of neurological conditions, including PD.

#### 2.1.1 CNS inflammation

Astrocytes play a dual role in CNS inflammation, exhibiting both pro-inflammatory and anti-inflammatory activities. They interact with other cells and molecules within the CNS by secreting various cytokines, chemokines, and neuroregulatory molecules [28,29]. This multifunctional capability highlights their impact on the neuroinflammatory landscape. Activated astrocytes are commonly detected in patients with PD, implicating neuroinflammation as a potential contributor to PD pathogenesis. Among the key regulators, NURR1 (NR4A2), an orphan nuclear receptor and a genetic risk factor for familial PD [30,31], plays a pivotal role in both the development of dopaminergic neurons and the suppression of inflammation in microglia and astrocytes. In astrocytes, NURR1 recruits CoREST corepressor complexes to NF-κB, facilitating the clearance of NF-κB-p65 and thereby reducing inflammation. Conversely, astrocytic NURR1 knockdown significantly increases the expression of toxic mediators, such as nitric oxide (NO) and reactive oxygen species (ROS), exacerbating disease pathology [32]. Besides, in the acute experimental autoimmune encephalomyelitis (EAE) B6 mouse model, a reduction in reactive astrocytes correlates with increased disease severity and heightened CNS inflammation [33]. Conversely, in the non-obese diabetic (NOD) mouse model of EAE, selectively reducing reactive astrocytes during the disease's chronic phase improves disease pathogenesis and reduces the recruitment and activation of microglia and monocytes [34]. These contrasting outcomes underscore the functional heterogeneity of astrocytes in CNS inflammation.

#### 2.1.2 Glutamate homeostasis

Glutamate is the predominant excitatory neurotransmitter in the CNS. However, its dysregulation can lead to excitotoxicity, a detrimental process resulting in neuronal damage and death. Astrocytes are central to the regulation of glutamate levels, effectively removing excess glutamate from the synaptic cleft through high-affinity glutamate transporters, namely excitatory amino acid transporter 1 (EAAT1) and EAAT2. These transporters are crucial for maintaining glutamate homeostasis, ensuring synaptic plasticity, and promoting neuronal survival [35]. The importance of this regulatory mechanism is particularly evident in the context of neurodegenerative diseases such as PD. In PD, the misfolded α-synuclein peptides in the brain have been shown to disrupt glutamate uptake by astrocytes through the inhibition of EAAT2 and EAAT1 [36,37]. This disruption leads to the overactivation of glutamate receptors in neurons, resulting in increased intracellular calcium (Ca^2+^) levels and subsequent neuronal dysfunction and degeneration [38].

#### 2.1.3 Ion homeostasis

Astrocytes regulate neuronal excitability by promoting the absorption and buffering of K^+^ in the brain [39,40]. This process, known as K^+^ spatial buffering, involves a variety of K^+^ channels expressed by astrocytes, which effectively remove excess K^+^ ions from the extracellular fluid, thereby preventing neuronal hyperactivity and maintaining the delicate balance of neural signals [41]. Dysfunction in astrocyte K^+^ channels can disrupt this critical regulatory mechanism, leading to an accumulation of K^+^ in the extracellular space. Such dysregulation can result in neuronal overexcitation, a condition that increases the risk of neuronal damage and contributes to the pathogenesis of neurodegeneration. In PD mouse model, astrocytic Kir6.1/K-ATP channel knockout inhibits mitophagy in astrocytes and promotes neuronal death via NF-κB-C3-C3aR astrocyte-neuron signaling [42,43]. In addition, disturbances in the regulation of extracellular glutamate levels by astrocytes can exacerbate this problem, further increasing the susceptibility to excitatory toxicity and neurodegenerative processes.

#### 2.1.4 Energy metabolism

Astrocytes are instrumental in fulfilling the energy requirements of neurons. This energy support is primarily facilitated through the astroglia-neuronal lactate shuttle, a mechanism wherein astrocytes metabolize glucose to lactate, which is then transferred to neurons as a vital energy source [44]. The release of lactate from astrocytes is triggered by various stimuli, including elevated levels of extracellular K^+^, increased glutamate uptake, and heightened intracellular Ca^2+^. These triggers ensure that neurons receive the energy substrates necessary to maintain their function and integrity. Dysfunctions in astrocyte metabolism, however, can have profound implications for neuronal health and are associated with the pathogenesis of various neurodegenerative diseases, such as multiple sclerosis (MS), Alzheimer's disease (AD), PD, and Huntington's disease (HD). These conditions may arise from or be exacerbated by impaired lactate transport from astrocytes to neurons, leading to energy deficits within neural circuits and contributing to neuronal dysfunction and degeneration.

#### 2.1.5 Neurotoxicity

**Fig. 2:**
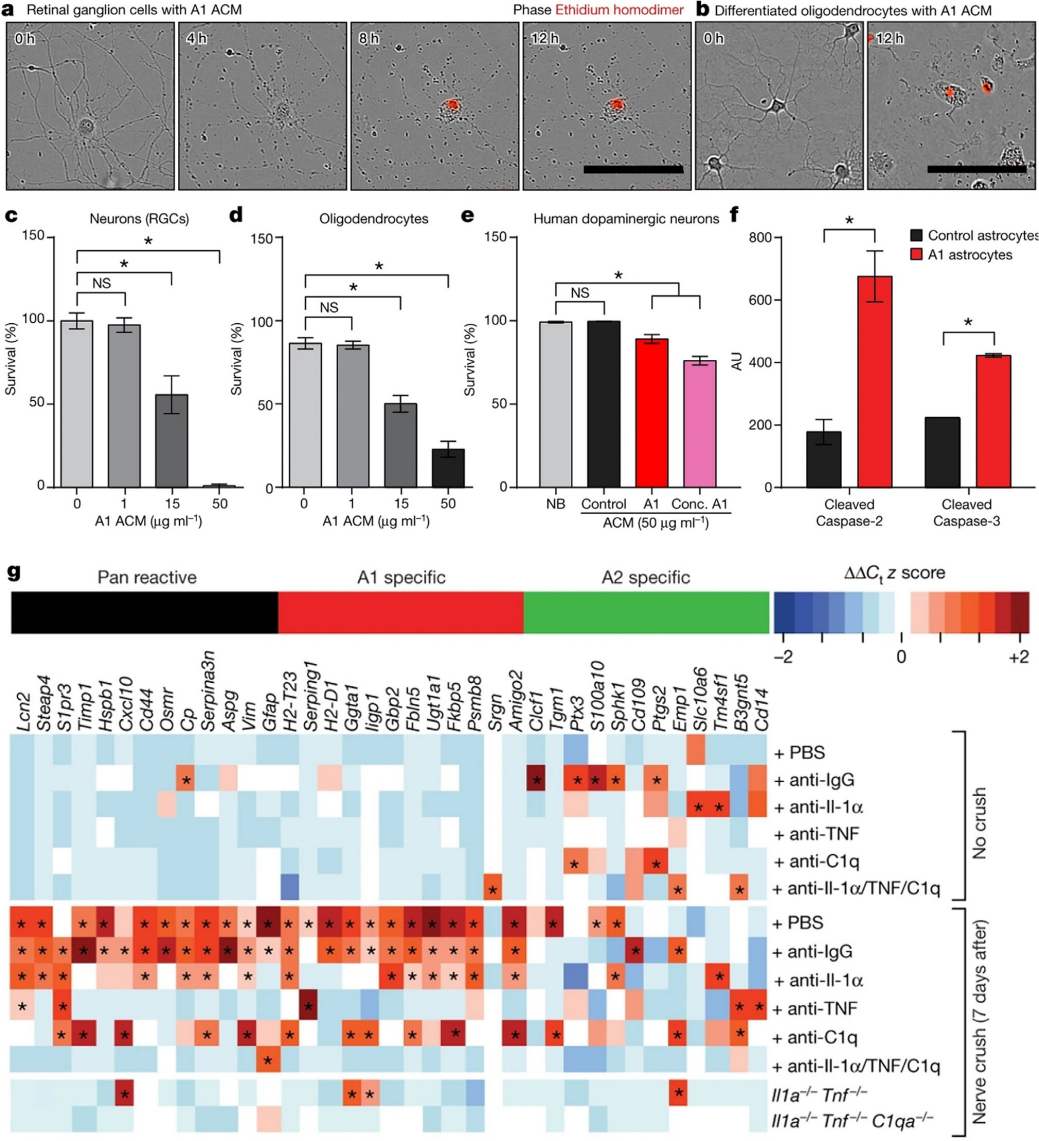
**Astrocyte-derived toxic factors inducing cell death.** (a–d) Representative phase images depict the death of purified retinal ganglion cells (RGCs) (a, stained with ethidium homodimer in red to indicate DNA in dead cells) and differentiated oligodendrocytes (b). Quantification of cell death in RGCs (c) and oligodendrocytes (d) after exposure to A1 astrocyte-conditioned medium (ACM) is shown. (e) Human dopaminergic neurons exposed to A1 ACM exhibit increased cell death, with quantification over 5 days. NB represents the neurobasal media control. (f) Western blot analysis demonstrates elevated levels of cleaved caspase-2 and caspase-3 in RGCs treated with A1 ACM, indicating apoptosis. (g) Retro-orbital optic nerve crush (ONC) induced A1 astrocytes in the retina. Intravitreal injections of neutralizing antibodies against IL-1α, TNF, and C1q blocked the production of A1 astrocytes. Reproduced from Ref. [18] with permission. Copyright 2017, Springer Nature.

Under certain conditions, astrocytes can contribute to neurotoxicity, adversely impacting neuronal survival. This neurotoxic activity is not uniform across all astrocytes but is attributed to specific subpopulations and is triggered by a complex interplay of factors. One such subpopulation involves C3^+^ neurotoxic astrocytes, which become harmful under the stimulation by complement factor C1q, tumor necrosis factor (TNF), and interleukin-1 alpha (IL-1α), all of which are produced by activated microglia [18]. These astrocytes have been implicated in inducing neuronal death, highlighting a critical pathway through which inflammation can escalate into neurodegenerative damage (Fig. 2). In PD, α-synuclein oligomers enhance the sensitivity of Toll-like receptor 4 (TLR4) expressed by astrocytes and microglia. This activation triggers the release of pro-inflammatory factors, including IL-1, IL-6, and TNF-α, which are primary drivers of neuronal death in PD [45]. Furthermore, the accumulation of α-synuclein aggregates within astrocytes has been implicated in neurodegeneration in mouse models. For instance, mutant mice overexpressing the

A53T α-synuclein variant specifically in astrocytes exhibits microglial activation, reactive astrocyte proliferation, and significant DA neuron loss, collectively promoting PD progression [46]. Postmortem analyses of brain tissues from PD patients further highlight the impact of α-synuclein accumulation. Aggregated α-synuclein in PD brains is associated with the infiltration of T cells. Astrocytes play a pivotal role in this immune response through antigen presentation and T cell activation. In PD brain tissue, perivascular and infiltrated CD4^+^ T cells are often surrounded by astrocytes expressing major histocompatibility complex class II (MHC-II), underscoring the crosstalk between astrocytes and T cells in PD pathogenesis [47]. Moreover, astrocytes can contribute to neurotoxicity through the secretion of long-chain saturated lipids [48]. These lipids, synthesized by the elongation of ultra-long chain fatty acids by elongase enzymes within astrocytes, are released in lipid granules and have been identified as neurotoxic factors. The presence of such lipids in the neural environment can exacerbate neuronal damage and death, further underscoring the dual nature of astrocytes in brain health and disease states.

### 2.2 Microglia

Microglia are mononuclear macrophages residing in the CNS. They originate primarily from mesodermal, neuroectodermal, and monocyte sources in blood circulation [49]. As a type of glial cell, microglia play crucial roles in supporting and protecting neurons, comprising 10% to 15% of the total glial cells in the CNS parenchyma [50]. They are widely distributed throughout the brain and spinal cord, with noticeable regional differences. The highest density of microglia is found in the midbrain substantia nigra, while the lowest density is in the brainstem and cerebellum [17]. As crucial immune cells in the brain, microglia form part of the inherent defense system of nervous tissue.

#### 2.2.1 Immune surveillance and defense

**Fig. 3:**
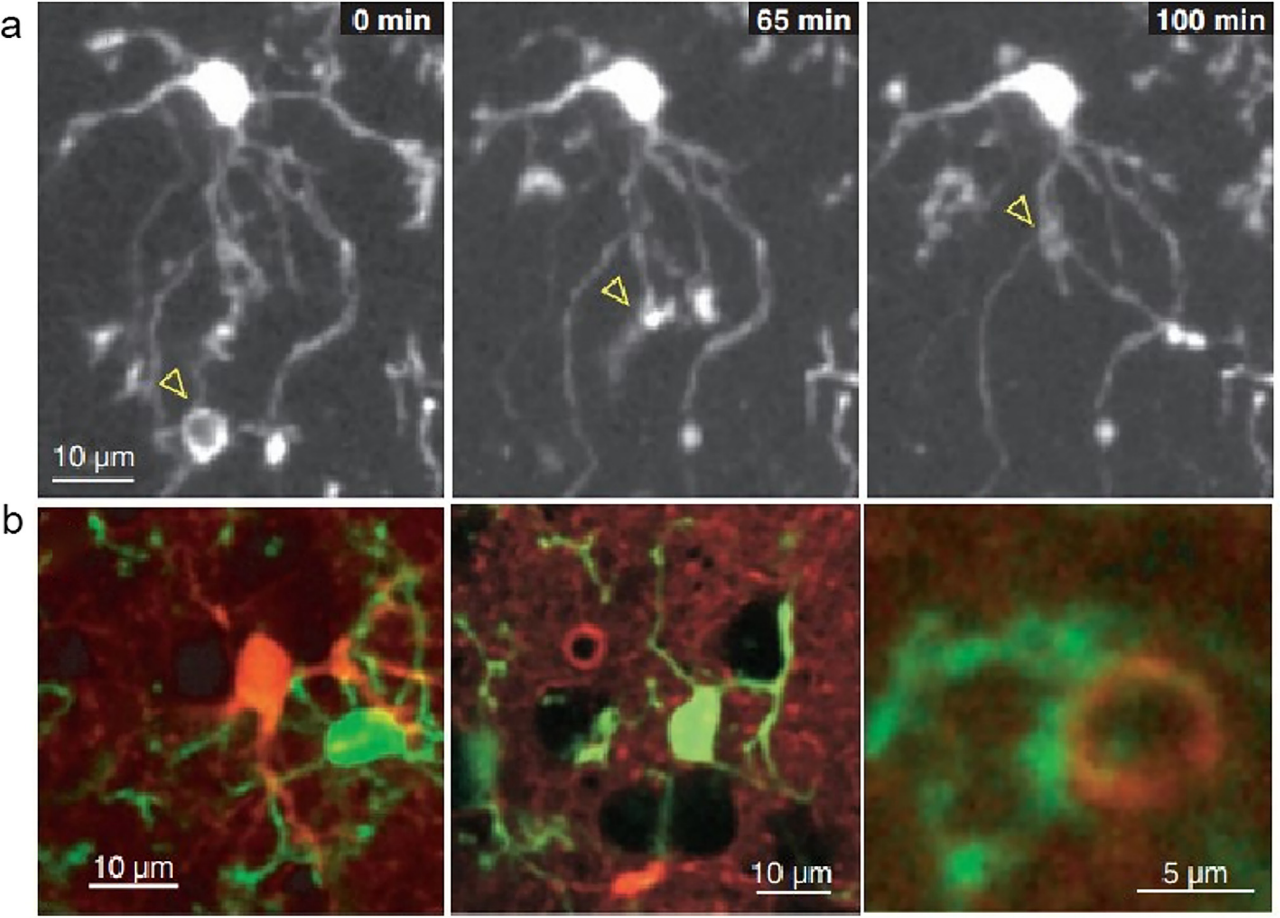
**Resting microglia engage in continuous environmental sampling and interaction with cortical elements.** (a) Time-lapse images demonstrate the spontaneous engulfment and subsequent evacuation of tissue components by microglial processes (highlighted by yellow arrowheads) over time (0 min, 65 min, and 100 min). (b) Microglial processes and protrusions interact with neighboring astrocytes (left), neuronal cell bodies (center, represented as dark unstained areas), and the astrocytic sheath surrounding a microvessel (right). Images are overlays of green-labeled microglia and red SR101 stain. Reproduced from Ref. [51] with permission. Copyright 2005, American Association for the Advancement of Science.

Microglia constantly monitor the nervous system environment to recognize and remove pathogens, such as bacteria and viruses, as well as damaged and dead nerve cells. They possess extensive filamentous pseudopods, which enable the cells to greatly increase their surface area, allowing them to continuously sample their surroundings and remove these pathogens and cell debris through phagocytosis [52]. This structure enables them to dynamically monitor the functional state of neighboring cells and sense molecular abnormalities. Various studies, using in vivo imaging in transgenic mice, have shown that microglia in the steady-state brain are never at rest but are constantly monitoring their tissue environment (Fig. 3) [51,53]. Mutations in the CSF1R gene, found in primary microglia, are further associated with the loss of neural networks and synaptic defects, ultimately leading to damage in the brain's white matter [54]. Therefore, continuous and effective microglial tissue monitoring is essential for the function of the adult human brain and for maintaining neural network homeostasis.

#### 2.2.2 Inflammatory response

In healthy adult brains, microglia play a synaptic pruning role, finely regulating synaptic connections and the function of neural circuits. At the same time, BDNF from microglia plays an important role in the formation of dendritic spines during learning [55,56]. However, under the stimulation of some pathological factors, microglia cells are activated into a pathological state, showing amoebia-like changes in morphology. Microglia rapidly activate and release inflammatory mediators, such as cytokines and chemokines, which help regulate the inflammatory response, attract other immune cells to injured or infected areas, and promote the repair of damaged tissue [49]. Activated microglia have historically been classified into two states: M1 (pro-inflammatory) or M2 (anti-inflammatory), and when these two states are out of balance, M1 microglia are associated with the production of pro-inflammatory cytokines, ROS, and NO, contributing to neuroinflammation and neuronal damage. Conversely, M2 microglia are involved in anti-inflammatory responses, tissue repair, and the release of neurotrophic factors [57]. However, recent research highlights that this binary classification oversimplifies the diverse functional states of microglia. Emerging evidence suggests that microglia exhibit a spectrum of activation states, including distinct disease-associated microglia (DAM) and neurodegeneration-associated phenotypes, characterized by unique transcriptomic and functional profiles. In the context of neurodegenerative diseases such as AD and PD, microglial activation is closely linked to the progression of pathological features such as amyloid plaques in AD and α-synuclein aggregation in PD. These phenotypes underscore the need to move beyond the M1/M2 framework to fully understand the roles of microglia in disease.

Recent evidence suggests that immune system dysfunction is intricately linked to the pathogenesis of PD. Neuropathological and neuroimaging studies have demonstrated the activation of inflammatory cells, alterations in inflammatory markers, and changes in immune cell populations in both the peripheral blood and cerebrospinal fluid of PD patients. Genetic associations between PD-causing genes and autoimmune diseases further underscore the connection between PD and immune dysregulation. A pivotal study in 1988 observed the presence of HLA-DR-reactive microglia in brain sections of PD patients. The quantity of these reactive microglia correlated with neuronal degeneration in the substantia nigra and striatopallidal pathway, marking an early link between innate immunity and PD [58,59]. Subsequent research has identified upregulated levels of monocytes and their precursor cells in the peripheral blood of PD patients [60]. In PD mouse models, C—C chemokine receptor 2 (CCR2)-positive peripheral monocytes infiltrated the substantia nigra. Suppressing CCR2 expression mitigated this infiltration and alleviated neuroinflammation, highlighting a critical role for peripheral immune cells in PD pathology [60,61]. Additionally, upregulated expression of major histocompatibility complex (MHC) molecules and infiltration of T cells have been documented in the substantia nigra and striatum of MPTP-induced PD mouse models. Activated microglia in PD brains contribute to the elevated levels of inflammatory markers, including IL-1β, TNF, IL-6, TGFβ, and ROS, in both central and peripheral compartments, such as the substantia nigra, striatum, cerebrospinal fluid, and peripheral blood. Advances in neuroimaging have provided valuable tools for assessing neuroinflammation in PD [62,63]. PET imaging using TSPO (translocator protein) ligands, such as ^11^C-PK11195, has shown increased microglial activation in PD patients compared to healthy controls. Although TSPO-based imaging has limitations due to its expression in other myeloid cells [64], newer ligands like [^11^C]DPA-713 have confirmed widespread microglial activation during the early stages of PD, implicating neuroinflammation as a pathogenic factor [65]. Genetic studies further strengthen the link between PD and immune dysfunction. Genes implicated in PD pathogenesis, such as LRRK2, SNCA, GBA, PRKN, and PINK1, are associated with immune regulation [66,67]. Moreover, shared genetic variations between PD and autoimmune diseases, such as Crohn's disease, point to overlapping mechanisms involving the immune system in PD development [68,69]. These findings collectively underscore the central role of neuroinflammation and immune dysregulation in PD and highlight the potential for targeting these pathways in therapeutic strategies.

#### 2.2.3 Regulation of neuronal function

Microglia are involved in the regulation of neurotransmission and neuronal activity through their interactions with neurons, affecting the function and plasticity of the nervous system [70]. Microglia support the survival and regeneration of neurons by releasing neurotrophic factors, which contribute to the repair and regeneration of the nervous system [71]. In some cases, they also promote synaptic remodeling between neurons and the reconstruction of neural networks. During development, microglia eliminate the apoptotic residues of excess newborn neurons through phagocytosis and have a catalytic effect on neurogenesis during brain development. Microglia affect the removal of damaged neurons by participating in the regulation of autophagy and apoptosis of neurons. In the self-protection mechanism of early AD, the production of Aβ by neurons activates microglia and astrocytes, which then trap and clear Aβ from the brain, while also releasing neuroprotective factors to safeguard the neurons [72]. In PD, this function of microglia may help clear damaged neurons, but it may also exacerbate neuron damage.

#### 2.2.4 Regulation of α-synuclein

During the development of Parkinson's disease, α-synuclein can be secreted into the extracellular space of neurons. Microglia are the primary cell type responsible for scavenging accumulated α-synuclein in the CNS [73]. Once in the extracellular environment, α-synuclein can be engulfed, degraded, or transformed into more pathogenic forms by microglia, eventually being released back into the extracellular space [74]. Different forms of α-synuclein, including oligomers and fibrils, enter microglia via receptor-mediated endocytosis, a key mechanism in its uptake both in vitro and in vivo. Extracellular α-synuclein oligomers, acting as damage-associated molecular patterns (DAMPs), activate toll-like receptor 2 (TLR2) on the surface of microglia. This activation triggers a cascade involving NF-κB and p38 MAPK signaling pathways, leading to the upregulation of neurotoxic substances, including cytokines, ROS, and NO [75,76]. Additionally, α-synuclein fibrils bind to Fc gamma receptor IIB (FcγRIIB), mediating raft-dependent internalization via downstream SHP-1/SHP-2 phosphorylation, further amplifying inflammatory responses [77,78].

Microglia utilize multiple pathways to degrade α-synuclein aggregates, including the autophagy-lysosome pathway (ALP) and the ubiquitin-proteasome system. A specific mechanism involves synaptic phagocytosis mediated by TLR4-NF-κB-p62 signaling. The autophagy receptor p62 enables microglia to engulf α-synuclein into autophagosomes, where it undergoes degradation via selective autophagy, termed “synuclein autophagy” [79].

Emerging evidence indicates that microglia can secrete α-synuclein in both membrane-bound and free forms via various pathways, including non-endoplasmic reticulum-dependent exocytosis, exosomes, and tunneling nanotubes (TNTs) [80]. The CNS environment facilitates extensive cellular crosstalk, with α-synuclein being transmitted between neurons, astrocytes, and pericytes [81]. For example, microglia exposed to α-synuclein can form TNTs to transfer α-synuclein to neighboring immature microglia, sharing the burden of aggregated α-synuclein degradation. Additionally, healthy microglia can donate intact mitochondria to α-synuclein-overloaded cells, reducing their inflammatory profile and cytotoxicity. This bidirectional transmission of α-synuclein and mitochondria via TNTs significantly attenuates the inflammatory response and neurotoxicity of microglia [82,83].

### 2.3 Oligodendrocytes

Oligodendrocytes in the CNS play a critical role in forming myelin sheaths, which are multi-layered fatty membranes that wrap around axons. These sheaths enable rapid and efficient electrical signal transmission while providing essential metabolic and nutritional support to neurons [84,85]. As non-proliferative cells, oligodendrocytes primarily arise from oligodendrocyte progenitor cells (OPCs), which are characterized by the expression of proteoglycan glial antigen (NG2) and platelet-derived growth factor receptor α (PDGFR-α) [86]. Present in both white and gray matter, OPCs constitute the largest dividing cell population in the adult CNS. Throughout their lifespan, they retain the capacity to proliferate and differentiate into mature oligodendrocytes, contributing significantly to the maintenance of periaxonal myelin and ensuring efficient neuronal conduction.

In the context of traumatic or non-traumatic diseases, demyelination prompts OPCs to migrate actively to lesion sites. Once there, they differentiate into mature oligodendrocytes, enclosing surviving axons to protect them from further degeneration and restore normal neuronal conduction [87,88]. For instance, in the 6-hydroxydopamine (6-OHDA) PD mouse model, anti-inflammatory treatments activate adult neural stem cells, resulting in the production of dentate glial cells. These cells stabilize axonal integrity and functionality, thereby mitigating motor impairments associated with basal ganglia dysfunction.

However, OPCs dysfunction or impaired differentiation exacerbates neurodegenerative diseases, including PD, multiple system atrophy, and Alzheimer's disease [[88], [89], [90], [91]]. β-glucocerebrosidase (GBA1), a lysosomal enzyme responsible for degrading gangliosides like glucocerebroside (GlcCer), plays a pivotal role in these processes. Mutations in the GBA1 gene result in lysosomal dysfunction [92,93], contributing to Gaucher disease (GD) and representing one of the most common genetic risk factors for PD [94]. Recent studies underscore the necessity of GBA1 activity for oligodendrocyte differentiation and proper myelination. Loss of GBA1 function in oligodendrocytes disrupts myelination, promotes α-synuclein accumulation, triggers astrogliosis, and induces early markers of neurodegeneration [95]. In synucleinopathies, axons with poor myelin are particularly susceptible to damage, with myelin loss strongly correlating with α-synuclein accumulation in oligodendrocytes [96]. Moreover, the absence of OPCs exacerbates neuroinflammation and neurodegeneration during CNS injuries [97]. In the MPTP mouse model, OPCs depletion intensifies dopaminergic neuron loss and enhances microglial reactivity [90].

Genome-wide association studies (GWAS) of substantia nigra and cortical tissues have revealed a genetic link between PD susceptibility and oligodendrocytes or OPCs, including genes such as MOBP [98]. Transcriptomic analyses further demonstrate that changes in oligodendrocyte and OPCs gene expression profiles can predict clinical outcomes in PD, highlighting their crucial role in the disease [99].

## 3 Molecular mechanisms of glial crosstalk in Parkinson's disease

**Fig. 4:**
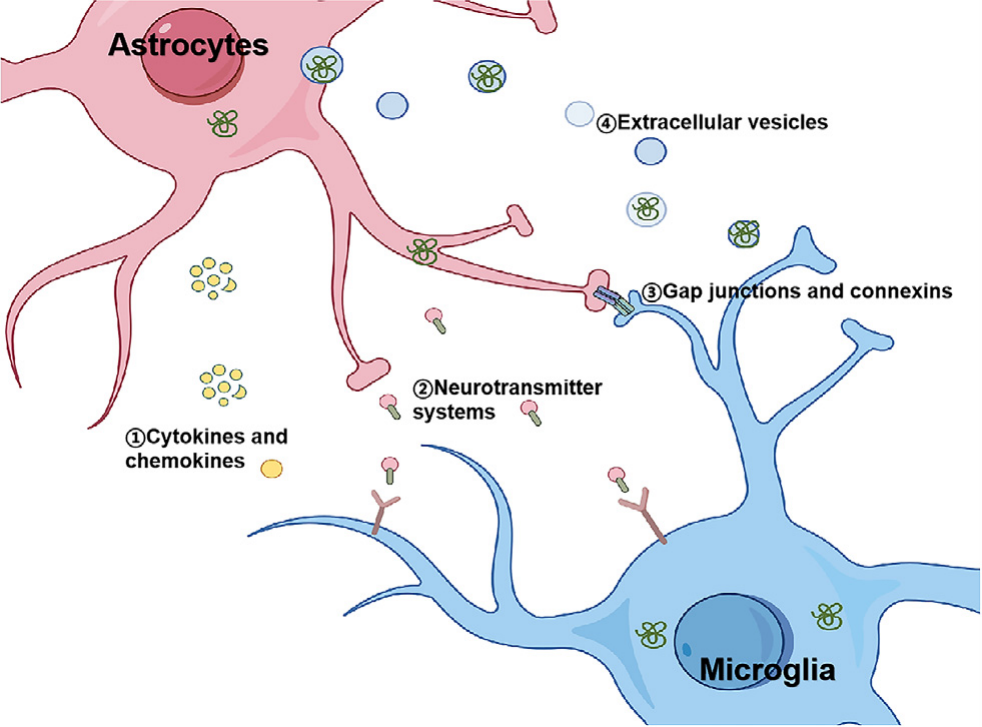
**Mechanisms of glial cell crosstalk in PD.** The dynamic interactions between astrocytes and microglia are mediated by several key mechanisms. These include (1) cytokines and chemokines, which promote or resolve neuroinflammation; (2) neurotransmitter systems, such as glutamate and ATP, which regulate glial activity and neuronal signaling; (3) gap junctions and connexins, facilitating direct intercellular communication; and (4) extracellular vesicles, which transfer proteins, RNA, and other bioactive molecules to modulate recipient cell function.

The interplay between glial cells is a key factor in regulating neuroinflammation and neurodegeneration in PD. Understanding the mechanisms that mediate this crosstalk offers critical insights into the progression of PD and provides potential therapeutic targets. These interactions are facilitated through a variety of pathways and molecular systems, each contributing to the dynamic and often pathological communication between glial cells. Below, we delve into four primary mechanisms that mediate glial cell interactions in PD: cytokines and chemokines, extracellular vesicles, gap junctions and connexins, and neurotransmitter systems (Fig. 4).

### 3.1 Cytokines and chemokines

Microglia in the CNS are responsible for monitoring the environment and responding to injury or pathogens. Astrocytes provide nutritional support to neurons, participate in the formation and maintenance of the blood-brain barrier, and play a crucial role in the repair process following neural injury [17,100]. Meanwhile, oligodendrocytes are primarily investigated for their role in the formation of myelin to ensure the conduction of nerve impulse [101]. During neuroinflammation, these cell types communicate with each other by releasing and responding to various cytokines and chemokines, collaboratively participating in the regulation of the inflammatory response [102,103].

**Fig. 5:**
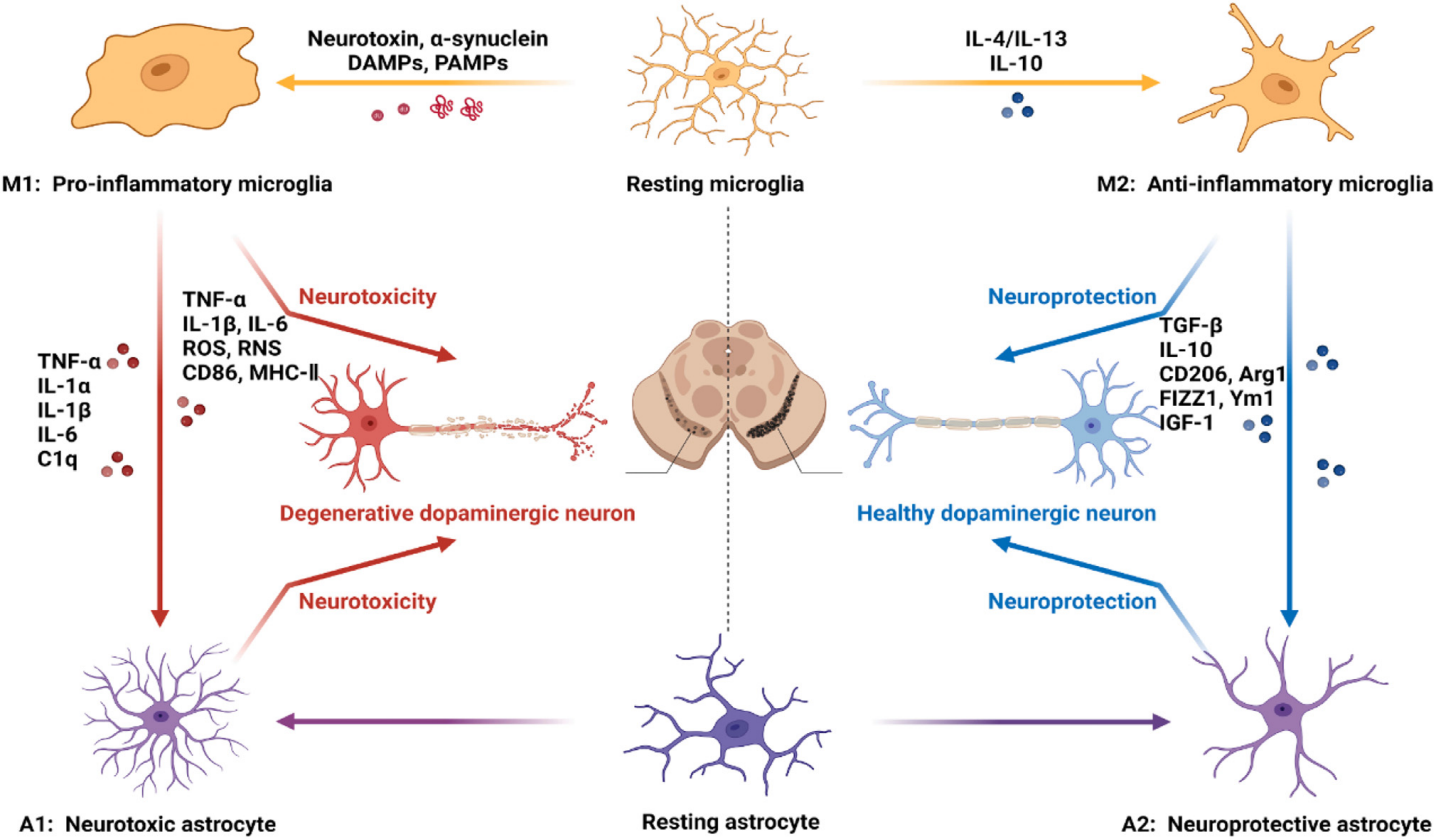
**Glial crosstalk between microglia and astrocytes though cytokines and chemokines.** This figure illustrates the bidirectional communication between microglia and astrocytes, mediated by cytokines and chemokines, and its impact on neuronal health. In a neuroprotective state, anti-inflammatory microglia (M2) release factors such as IL-4, IL-10, and IL-13, which promote the activation of neuroprotective astrocytes (A2). This interaction supports neuronal survival and repair through the secretion of TGF-β, IL-10, and IGF-1 by A2 astrocytes. Conversely, in pathological conditions, pro-inflammatory microglia (M1) are activated by neurotoxins, α-synuclein, and DAMPs/PAMPs, leading to the release of pro-inflammatory cytokines (TNF-α, IL-1β, IL-6, and C1q). These cytokines activate neurotoxic astrocytes (A1), which in turn exacerbate neuroinflammation and induce neurodegeneration. This crosstalk between M1 microglia and A1 astrocytes contributes to the progressive loss of dopaminergic neurons seen in diseases such as Parkinson's disease.

Microglia, when activated by DAMPs or pathogen-associated molecular patterns (PAMPs), release a variety of pro-inflammatory cytokines such as TNF-α, IL-1β, and chemokines like CCL2 [104]. These signaling molecules not only amplify the inflammatory response but also activate surrounding microglia and astrocytes, creating a feedback loop that can perpetuate neuroinflammation [105,106]. Astrocytes, in turn, respond to these signals by releasing their own cytokines and chemokines, such as interleukin-6 (IL-6) and CCL2, further amplifying the inflammatory response and attracting more immune cells to the site of injury or infection [28,107,108]. This bidirectional communication between microglia and astrocytes is crucial for the coordination of the CNS's immune response but can also lead to chronic inflammation if dysregulated (Fig. 5). In PD, the chronic activation of microglia and astrocytes and their sustained release of pro-inflammatory cytokines and chemokines contribute significantly to the neurodegenerative process. The overproduction of these inflammatory mediators can disrupt neuronal homeostasis, cause synaptic dysfunction, and eventually lead to neuronal death. For example, Liddelow and colleagues [18] reported that LPS-activated microglia induce a neurotoxic phenotype in reactive astrocytes. Specifically, they found that IL-1α, TNF-α, and C1q secreted by microglia induce a transcriptional response in astrocytes. This response is characterized by the production of an unidentified neurotoxic factor, reduced phagocytic activity, and decreased expression of neurotrophic factors [109]. Complement component 3 (C3) is considered a marker for these neurotoxic astrocytes. Based on the expression of C3, researchers have identified this subpopulation of neurotoxic astrocytes in several neurodegenerative diseases, such as Huntington's disease, Alzheimer's disease, and multiple sclerosis [110]. This finding suggests a common mechanism mediating microglia-astrocyte crosstalk in these conditions. In PD mouse models induced by LPS or MPTP, microglial NADPH oxidase (NOX2)-derived H_2_O_2_ promotes astrocyte proliferation through the STAT1/3-dependent pathway. Inhibiting or downregulating NOX2 activity in these models has been shown to suppress microglial activation, thereby alleviating astrocyte hyperplasia. This evidence suggests that NOX2-derived H_2_O_2_ in PD serves as a direct signal modulating microglia-astrocyte crosstalk, contributing to neuroinflammatory processes [111].

Astrocytes play a dual role in regulating oligodendrocytes and myelination. On the one hand, astrocytes secrete various growth and trophic factors, such as insulin-like growth factor 1 (IGF-1), leukemia inhibitory factor (LIF), and fibroblast growth factor-2 (FGF-2), which promote oligodendrocyte survival and differentiation, thereby enhancing myelin formation [[112], [113], [114]]. On the other hand, astrocytes can contribute to pathological outcomes during CNS injury, including PD. Reactive astrogliosis, characterized by astrocyte proliferation and hypertrophy, aims to minimize tissue damage and restore homeostasis. However, the glial scar formation resulting from reactive astrogliosis can impede the access of OPCs to lesion sites, thus preventing effective myelin regeneration [115]. In addition, astrocytes can inhibit OPCs differentiation and hinder myelin repair by secreting pro-inflammatory and regulatory molecules, including IFN-γ, TNF-α, and PDGF-α [116,117]. Astrocyte-oligodendrocyte interactions are crucial determinants for successful myelin regeneration. For instance, in a male mouse model with focal lesions, persistent activation of the Nrf2 pathway in astrocytes led to dysregulation of the cholesterol biosynthesis pathway, ultimately impairing oligodendrocyte survival and myelin regeneration. Pharmacological inhibition of Nrf2 using luteolin or stimulation of cholesterol biosynthesis/excretion improved oligodendrocyte survival, underscoring the regulatory role of astrocytes in cholesterol transport and myelin repair [118]. OPCs also contribute to CNS homeostasis by releasing transforming growth factor-beta 2 (TGF-β2), which plays a key role in maintaining microglial homeostasis. TGF-β2 enhances the expression of CX3CR1 on microglia, thereby promoting their neuroprotective phenotype. Conversely, ablation of NG2-glial cells, which are closely related to OPCs, has been shown to induce neuronal cell death and microglial activation in the hippocampus [119].

### 3.2 Extracellular vesicles

Astrocytes and microglia interact in the nervous system through various mechanisms, with extracellular vesicles (EVs) playing a crucial role in this interaction. EVs, including exosomes and microvesicles, are lipid bilayer vesicles released by cells that contain various molecules, such as mRNA and miRNA, which regulate the functions of recipient cells [[120], [121], [122]].

Research has shown that EVs are essential for intercellular communication in the nervous system. Astrocytes, the most abundant glial cells in the brain, can take up EVs from other cell types, which can then modulate their function. For example, EVs derived from neurons can increase the expression of the glutamate transporter GLT1 in astrocytes through miR-124a, thereby maintaining the homeostasis of glutamate concentrations in the synaptic cleft [123]. The role of EVs under pathological conditions has also been extensively studied. Neuron-derived EVs have been found to mediate the aggregation of α-synuclein, a hallmark of PD [124]. This aggregation not only contributes to the pathology of PD but also highlights the role of EVs in spreading disease-related proteins and exacerbating neurodegenerative processes.

In PD, the misfolded protein aggregate α-synuclein can be transmitted via EVs, facilitating its propagation between neurons as well as between neurons and glial cells. This intercellular transfer accelerates the progression of PD pathology [125]. Studies have demonstrated that SH-SY5Y cells secrete exosomes containing α-syn, and its secretion into exosomes is mediated through a calcium-dependent endosomal mechanism [126]. The quality and quantity of EVs are regulated by the ALP. Dysfunction in ALP, induced by modulators like bafilomycin, promotes the release and dissemination of exosomal α-synuclein to neighboring cells, exacerbating pathological spread [127]. Furthermore, recent evidence indicates that α-synuclein can stimulate microglia to produce exosomes. For instance, when microglia are treated with preformed fibrils (PFF) of α-synuclein, oligomeric α-synuclein is packaged into microglia-derived exosomes. These exosomes can subsequently infect healthy neurons, inducing pathological protein aggregation in recipient cells [128,129]. Additionally, α-synuclein interacts with the purinergic P2X7 receptor (P2X7R) on the surface of microglial cells, activating the P2X7R/PI3K/AKT signaling pathway. This activation enhances the release of secretory cathepsin L (CTSL) via exosomes, leading to neuronal damage and death. Specific antagonists targeting this pathway, such as the exosome release inhibitor GW4869 and P2X7R knockdown strategies, have been shown to significantly reduce cortical neuron damage in mice, highlighting the therapeutic potential of modulating this mechanism [130].

Microglia, as the resident macrophages of the brain, can both release and take up EVs, thereby playing a significant role in the immune responses of the nervous system. In a multiple sclerosis model, EVs released by microglia and taken up by astrocytes contribute to the maturation of OPCs, aiding in myelin regeneration [131]. Additionally, ATP-stimulated microglia-derived EVs (ATP-EVs) are taken up by astrocytes, leading to increased contact time between ATP-EVs and astrocyte surfaces, which in turn upregulates the expression of inflammatory cytokines such as IL-1β, IL-6, and TNF-α in astrocytes [132]. Additionally, in ischemic stroke, microglia-released EVs can exert beneficial effects on OPCs. The EVs from pro-regenerative microglia contain trans-membrane TNF (tmTNF), which enables the EVs to restore the degradative activity of receptor microglia, promote OPCs differentiation and function, accelerate oligodendrocyte maturation and myelin regeneration, thereby assisting functional recovery in ischemic mice [133]. EVs function not only under disease conditions but also play a role in the regulation of cellular functions in a healthy brain. For instance, Lopez-Ramirez, Miguel Alejandro et al. found that EVs derived from monocytes can be taken up by astrocytes and potentially affect blood-brain barrier (BBB) permeability through miR-155 [134]. The complex interaction between astrocytes and microglia through EVs is essential for maintaining normal nervous system function and significantly impacts the development and progression of neurological diseases.

### 3.3 Gap junctions and connexins

Direct cellular communication in the CNS involves gap junctions and TNTs. Gap junctions are channels that allow ions and small molecules to transfer directly between cells [135]. These connections are formed by the docking of two half-channels, each contributed by one of the adjacent cells. Each half-channel is composed of six protein subunits called connexins, which differ in type and affect the selectivity and permeability of the gap junctions [136]. Connections between astrocytes and microglia can also form heterotypic gap junctions, promoting the direct exchange of cytoplasm and coordination of cell activities. Astrocytes express various connexins, including Cx43 and Cx30, which are critical for forming gap junctions between astrocytes and potentially with other glial cells [137,138]. Moreover, Cx32 plays a crucial role in the selective uptake and propagation of α-synuclein oligomers in neurons and oligodendrocytes. In models simulating PD and MSA, as well as in transgenic mice, the upregulation of Cx32 was observed as a result of α-synuclein overexpression. This upregulation facilitated the uptake and intercellular transfer of α-synuclein assemblies between neurons and oligodendrocytes. Notably, the use of gap junction inhibitors such as carbenoxolone (CBX), mefloquine (MQ), and 2-aminoethoxydiphenyl borate (2-APB) successfully blocked these processes, thereby preventing the spread of α-synuclein aggregates in PD and MSA models [139].

Although microglia are traditionally considered less involved in forming gap junctions, evidence suggests that under certain conditions, they can participate in gap junctional communication, directly or indirectly influencing the astrocyte network.

Gap junctions between astrocytes form a functional syncytium, enabling the distribution of metabolic substrates, ions, and signaling molecules across large areas. This network is vital for maintaining the homeostasis of the brain's extracellular environment [140,141]. It also plays a crucial role in the propagation of calcium waves, allowing astrocytes to communicate over both short and long distances, which can influence microglial activation and function.

TNTs are thin membrane-like tubes that connect distant cells and allow the transfer of cellular components, including organelles, ions, and signaling molecules. TNTs promote communication between astrocytes and microglia and are implicated in various aspects of CNS function and pathology. They facilitate intercellular communication, helping coordinate responses to stress, injury, or infection. For example, the transfer of mitochondria from astrocytes to neurons via TNTs has been proposed as a mechanism to support neuronal survival under stress conditions [142]. In PD, TNTs can connect the cytosol of neurons and astrocytes, thus promoting the bidirectional transfer of α-synuclein. In addition, astrocytes can also connect to other glial cells based on the physical properties of these nanotubes, allowing α-synuclein to be transferred between different glial cells [142,143]. Similarly, TNTs between glial cells can facilitate the spread of inflammatory signals or neuroprotective factors [47].

### 3.4 Neurotransmitter systems

Astrocytes and microglia communicate through various neurotransmitter systems, significantly influencing brain homeostasis and function. Astrocytes play a crucial role in the uptake and recycling of neurotransmitters such as glutamate and GABA, thereby regulating synaptic transmission and preventing excitotoxicity. Glutamate, the primary excitatory neurotransmitter in the CNS, is taken up by astrocytes through excitatory amino acid transporters (EAATs), converted into glutamine, and then supplied back to neurons. This glutamate-glutamine cycle is essential for maintaining synaptic transmission and overall brain function.

Astrocytes also release glutamate, which can act on microglia through various glutamate receptors, including metabotropic and ionotropic receptors such as NMDA and AMPA receptors [144]. Glutamate signaling can modulate microglial activity, influencing their proliferation, migration, and release of pro-inflammatory or anti-inflammatory factors [145,146]. However, excessive glutamate release from astrocytes can contribute to excitotoxicity and exacerbate inflammation.

ATP signaling is another critical pathway in astrocyte-microglia communication. ATP released by astrocytes can activate microglia, promoting the release of cytokines and chemokines. Conversely, adenosine, a breakdown product of ATP, typically acts as an anti-inflammatory signal, suppressing microglial activation and promoting the resolution of inflammation [147,148]. GABA, the primary inhibitory neurotransmitter in the CNS, can also be released by astrocytes. GABA then interacts with GABA receptors on microglia, exerting an inhibitory effect on microglial activation and reducing the release of pro-inflammatory mediators [149]. This GABAergic signaling from astrocytes helps to balance excitatory and inhibitory signals in the brain, contributing to the regulation of neuroinflammation.

### 3.5 Targeting glial cells in treatment strategies

During the development of the nervous system, microglia help shape neural circuits by regulating synaptic biomolecules and pruning synapses. They play a monitoring role by constantly sensing changes in the surrounding environment through the expansion and contraction of their tentacles [150,151]. When the CNS is damaged, microglia assume a phagocytic role, engulfing and eliminating microorganisms, dead cell debris, protein aggregates, and soluble antigens that may harm the CNS [152]. However, under conditions of stimulation or neuroinflammation, or when microglia are dysfunctional, they may also damage and kill neurons through phagocytosis or the secretion of inflammatory factors, contributing to the occurrence and development of neurodegenerative diseases such as PD [153]. Astrocytes regulate glutamate and ion homeostasis, cholesterol and sphingolipid metabolism, and respond to environmental factors, all of which have been implicated in neurological diseases. Astrocytes also exhibit significant heterogeneity, with developmental programs and stimulus-specific cellular responses regulating their location and cell-cell interactions in the CNS.

Targeting microglia, astrocytes, and their interactions offers significant therapeutic potential for neurological diseases. Modulating microglial activation can reduce neuroinflammation, while enhancing astrocytic functions can prevent excitotoxicity and improve neuroprotection. Additionally, focusing on their cross-talk, such as through extracellular vesicles, presents novel approaches to maintaining CNS homeostasis and treating neurodegenerative disorders.

### 3.6 Modulating microglial activation

Modulating microglial activation is a promising strategy for reducing neuroinflammation and protecting neurons in PD. Various therapeutic approaches include the use of non-steroidal anti-inflammatory drugs (NSAIDs), Toll-Like Receptor (TLR) inhibitors such as candesartan cilexetil, rifampin, TAK-242, and RSLA [[154], [155], [156], [157]], and endocannabinoid receptor agonists like JWH133 [158]. Additionally, inhibitors targeting the NF-κB and NLRP3 pathways, such as tanshinone I, α-asarone, JNJ7777120, and MCC950, as well as PPAR-γ agonists like pioglitazone and rosiglitazone, have shown potential in decreasing microglial activation and the release of inflammatory factors [[159], [160], [161], [162], [163], [164]]. The pro-inflammatory M1 phenotype of microglia is primarily induced by IFN-γ through activation of the JAK/STAT signaling pathway. Targeting this pathway or suppressing its downstream pro-inflammatory cytokines, such as TNF-α, IL-1β, and IFN-γ, has shown promise in alleviating neuroinflammation and improving PD outcomes [165]. In PD animal models, TNF-α has been effectively targeted through various strategies to mitigate M1 microglial toxicity. For example, in a 6-OHDA-induced PD rat model, a single injection of a lentivirus expressing dominant-negative TNF (DN-TNF) into the substantia nigra reduced dopaminergic neuron loss and ameliorated behavioral abnormalities [166]. Similarly, in the MPTP mouse model, treatment with the PPARγ agonist rosiglitazone protected neurons in the striatum and substantia nigra pars compacta by reducing microglial activation, suppressing TNF-α and iNOS production, and preventing nitric oxide-mediated toxicity [[167], [168], [169]]. Tanshinone-I, another compound, effectively reduced the production of pro-inflammatory mediators, such as nitric oxide and TNF-α, in BV2 microglial cells, thereby attenuating M1 microglial neurotoxicity [170]. Furthermore, the NLRP3 inflammasome inhibitor MCC950 significantly suppressed inflammasome activation, leading to improved motor performance in PD mouse models [171].

Beyond reducing microglial activation, promoting the anti-inflammatory M2 phenotype is crucial for mitigating neuroinflammation. This can be achieved by increasing the expression of anti-inflammatory factors through methods such as viral overexpression of IL-10 in the brain, or by promoting the transformation of microglia from the pro-inflammatory M1 state to the anti-inflammatory M2 state using agents like vitamin D and glatiramer acetate [172,173]. While these strategies show promise, further research and development are needed to advance them to clinical trials and develop effective treatments for PD and other neurodegenerative diseases involving microglial activation and neuroinflammation.

### 3.7 Enhancing astrocytic functions

Astrocytes play diverse roles in the CNS, secreting various cytokines, chemokines, and neuroregulatory molecules. Their functions can either exacerbate or mitigate disease severity, as evidenced by studies in mouse models. For instance, reducing reactive astrocytes increased disease severity and CNS inflammation in an acute EAE B6 mouse model [174], while selectively reducing reactive astrocytes during chronic progression improved disease pathogenesis and limited the activation of microglia and monocytes in NOD mouse EAE models [175]. This functional heterogeneity suggests that astrocytes can have both beneficial and harmful roles in neurodegeneration, including PD.

**Fig. 6:**
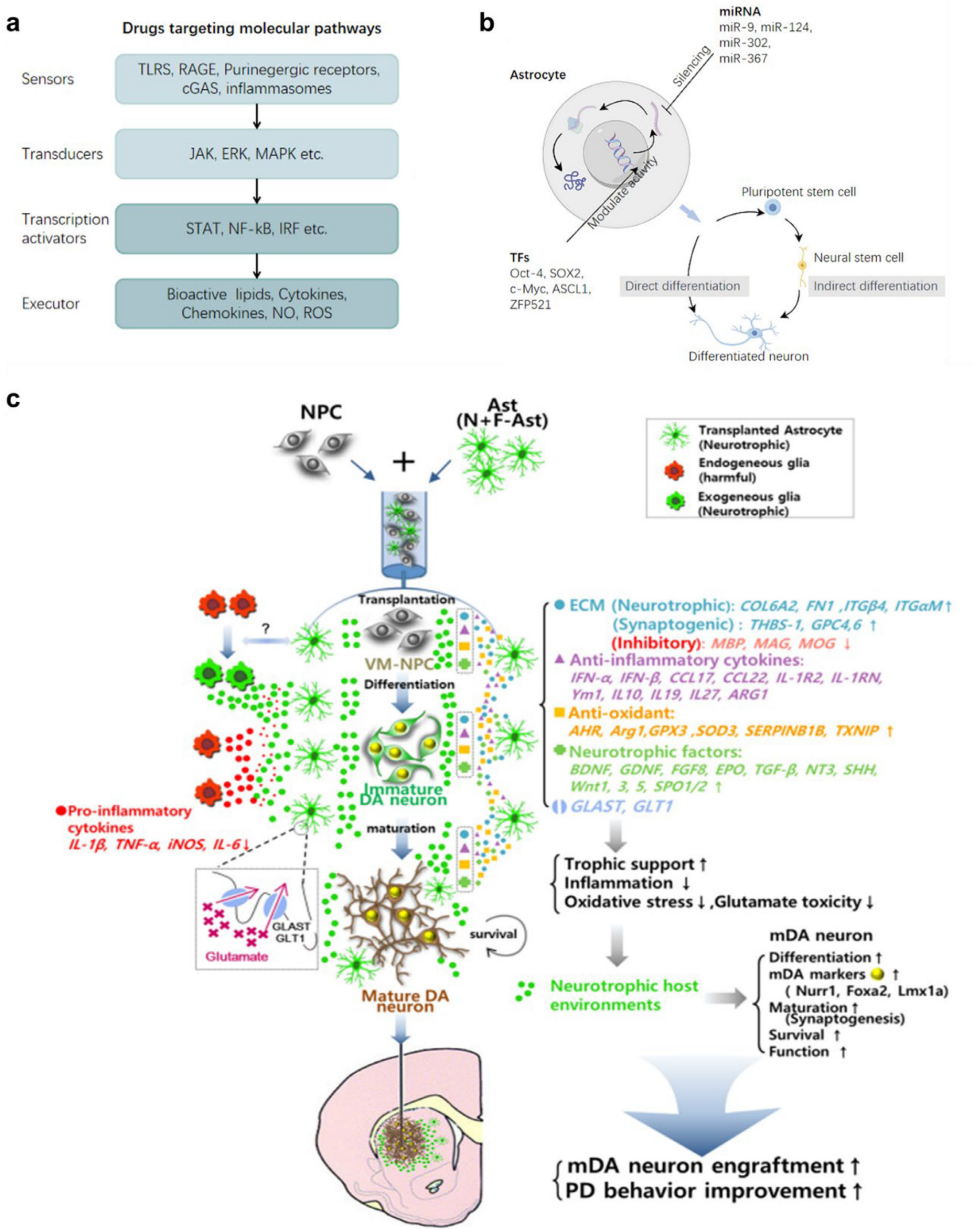
**Therapeutic** s**trategies for Parkinson's Disease through** m**odulating** a**strocyte** f**unctions.** This figure highlights various treatment strategies aimed at leveraging astrocyte functions to support neuronal survival and regeneration in PD. (a) Pharmacological approaches: Drugs targeting molecular pathways in astrocytes involve sensors (TLRs, RAGE, inflammasomes), transducers (JAK, ERK, MAPK), transcription activators (STAT, NF-κB, IRF), and executors such as cytokines, chemokines, and ROS. These pathways can be modulated to enhance neuroprotection and reduce inflammation. (b) Astrocyte reprogramming: Astrocytes can be reprogrammed into functional neurons using transcription factors (e.g., Oct-4, SOX2, c-Myc) or miRNAs (e.g., miR-9, miR-124). This approach can directly or indirectly generate neurons that contribute to neural regeneration in PD. (c) Co-grafting of Astrocytes in Cell Therapy for PD: Astrocytes (VM, N + *F*-VM-Ast) co-grafted with ventral midbrain neural progenitor cells (VM-NPCs) enhance survival, differentiation, and maturation of transplanted neurons. These astrocytes correct hostile brain environments by secreting neurotrophic extracellular matrix proteins (e.g., COL6A2, FN1), anti-inflammatory cytokines (e.g., IL-10, TGF-β), antioxidants (e.g., AHR, SOD2), and neurotrophic factors (e.g., BDNF, GDNF). They also promote glutamate clearance through GLAST/GLT1, reducing excitotoxicity. Ultimately, this co-grafting improves PD-related behaviors and enhances the engraftment of mature, functional dopaminergic neurons expressing midbrain-specific markers, thus supporting synaptic integration and neuronal survival. Reproduced from Ref. [178] with permission. Copyright 2018, American Society for Clinical Investigation.

Multiple therapeutic strategies targeting astrocytes in neurodegenerative diseases have been proposed. These strategies include pharmacological approaches targeting specific pathways in astrocytes (Fig. 6a), genetic modifications to alter the expression of specific genes or proteins (Fig. 6b), and transplantation of healthy astrocytes into affected areas. Additionally, transforming astrocytes into neurons to replace damaged cells has shown potential [176,177]. The mechanisms of action for these treatments include upregulating antioxidant molecules or beneficial cytokines, enhancing the GLT-1 function in anoxic environments, downregulating inflammatory mediators, and modulating the proliferation of reactive astrocytes (Fig. 6c) [178]. For example, drugs like EE promote astrocyte proliferation, while TSA, rapamycin, and paeonol inhibit it [179]. Other compounds, such as carnosine and overexpression of TIGAR and PAX6, enhance astrocyte function [180], whereas amlexanox and IP3R2 knockout inhibit it [181]. These studies underscore the dual nature of astrocytes in PD, highlighting the need for targeted therapies that can modulate their activity to achieve therapeutic benefits.

### 3.8 Modulating glial crosstalk

Enhancing the beneficial interactions between microglia and astrocytes can help resolve neuroinflammation and support neuronal survival. This can be achieved by promoting the release of anti-inflammatory and neurotrophic factors from these cells. Certain forms of cell therapy or gene therapy aimed at delivering anti-inflammatory cytokines or enzymes that degrade pro-inflammatory mediators can support this strategy [182,183].

**Fig. 7:**
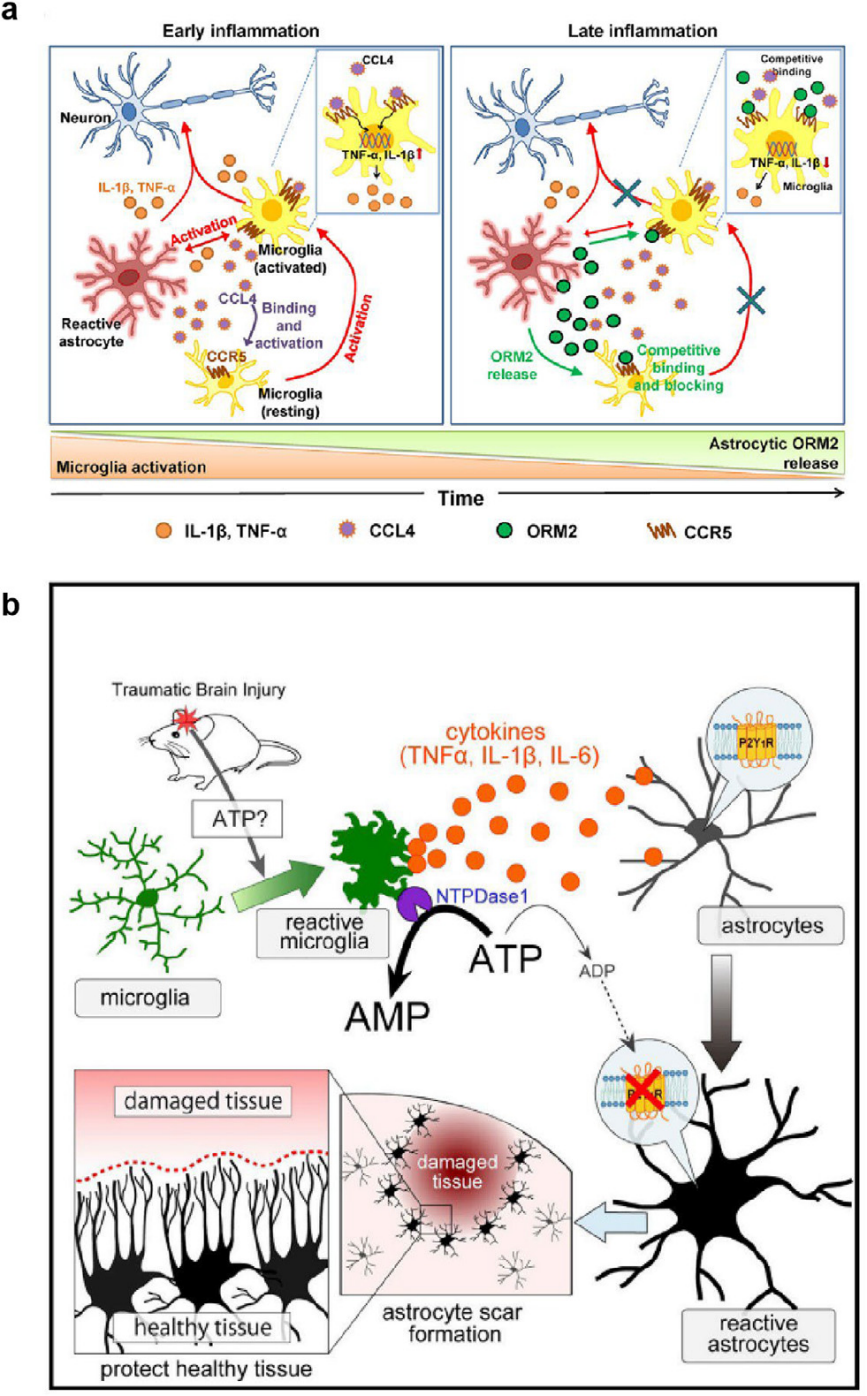
**Therapeutic strategies for modulating glial crosstalk in neuroinflammation.** (a) Schematic representation of the functions of ORM2 during neuroinflammation. In early inflammation, reactive astrocytes and activated microglia release pro-inflammatory cytokines (e.g., IL-1β, TNF-α) and chemokines such as CCL4, which binds to CCR5 receptors on microglia. This crosstalk further activates microglia, promoting their migration to inflammatory sites, potentially causing neuronal damage. During late inflammation, astrocytes release Orosomucoid 2 (ORM2), which competes with CCL4 for CCR5 binding, reducing microglial activation and migration, thus mitigating the inflammatory response and protecting neurons. Reproduced from Ref. [185] with permission. Copyright 2017, The Society for Neuroscience. (b) In response to brain trauma, microglia release ATP, which is converted to AMP through the NTPDase1 enzyme pathway. This signaling process downregulates P2Y1 receptors on astrocytes, transforming them into a reactive, neuroprotective phenotype. The astrocytic response helps protect healthy tissue by forming a scar that isolates damaged regions, limiting the spread of injury. Reproduced from Ref. [186] with permission. Copyright 2017, Elsevier.

Current research predominantly focuses on targeting the interactions between microglia and astrocytes, with several key ligands receiving widespread attention for their pivotal roles. Orosomucoids (ORMs), a group of small molecular-binding proteins known for their immunomodulatory functions, have been identified as significant in this context [184]. ORM2 proteins are primarily expressed and secreted by astrocytes in response to inflammatory stimuli. Jo Myungjin et al. found that during advanced stages of inflammation, ORM2 released by astrocytes binds to C—C chemokine receptor type 5 (CCR5), further regulating the activation of microglia and playing an anti-inflammatory role [185]. This interaction positions ORM2 as a potential target for anti-inflammatory therapy (Fig. 7a). In addition to immunomodulation, the communication between astrocytes and microglia also involves energy metabolism mechanisms mediated by ATP and its derivatives. Previous studies have discovered that activated microglia can release ATP, which in turn activates the reactive phenotype of astrocytes and reduces neuronal damage through the activation of astrocytic P2Y1 receptors (P2Y1R) (Fig. 7b) [186]. This synergy between astrocytes and microglia is crucial in regulating neuronal function in neurodegenerative diseases like PD. In the α-synuclein PFF-induced PD mouse model, activated microglia stimulated by α-synuclein PFF specifically induce the conversion of astrocytes into neurotoxic A1 astrocytes. This transformation is mediated by the release of pro-inflammatory factors, including IL-1α, TNFα, and C1q. NLY01, a long-acting GLP1R agonist with effective blood-brain barrier permeability, has demonstrated neuroprotective effects by inhibiting microglia-derived factors. By blocking the transformation of astrocytes into the A1 neurotoxic phenotype, NLY01 offers a promising therapeutic strategy for mitigating neurodegeneration in PD [187].

By targeting these various aspects of glial cell function and their interactions, it may be possible to develop more effective treatments for PD and other neurodegenerative diseases. This comprehensive approach offers hope for preserving neuronal health and improving clinical outcomes in patients with CNS disorders. Further research into the interactive networks of astrocytes and microglia will likely reveal more specific targets for action, enabling the development of targeted therapies to modulate neural functions and restore the activity of damaged neurons.

## 4 Conclusion and future perspectives

Neuroinflammation plays a critical role in the progression of PD. The activation and interactions of microglia and astrocytes are central to this process. Inhibiting the over-activation of these glial cells and their mediated neuroinflammation can effectively slow the progression of PD. However, the specific effects of microglial heterogeneity on PD pathology and the mechanisms driving the M1 and M2 states of microglia remain unclear. Further research into the key molecules and pathways involved in glial cell overactivation will provide new targets and strategies for PD treatment and drug development.

Advancements in high-throughput molecular characterization techniques will significantly enhance our understanding of glial cell heterogeneity and interactions within the CNS. By mapping glial cell subpopulations in different brain regions and disease states, we can answer critical questions about glial communication, the time scale of these interactions, and their malleability. Additionally, understanding microglia and astrocyte networks at a holistic level through functional and structural connectivity studies is essential. These approaches will illuminate how glial cells interact with neurons, other glial cells, and immune cells in health and disease, transforming our understanding of neural circuits.

In conclusion, the future of glial biology depends on integrating multiple high-throughput technologies to determine the location, plasticity, connectivity, regulation, and function of astrocyte subpopulations throughout the CNS. These comprehensive analyses will reveal key molecules and pathways involved in PD, providing novel targets and strategies for treatment and drug development. By advancing our understanding of glial cell interactions and functions, we can develop more effective therapies to combat neurodegenerative diseases like PD.

## Declaration of competing interests

The authors declare that they have no conflicts of interest in this work.
